# Dataset exploited for the development and validation of automated cyanobacteria quantification algorithm, ACQUA

**DOI:** 10.1016/j.dib.2016.06.042

**Published:** 2016-06-29

**Authors:** Emanuele Gandola, Manuela Antonioli, Alessio Traficante, Simone Franceschini, Michele Scardi, Roberta Congestri

**Affiliations:** a University of Rome Tor Vergata, Department of Biology, Via della Ricerca Scientifica 1, 00133 Rome, Italy; b Department of Mathematics, University of Rome Tor Vergata, Via della Ricerca Scientifica 1, 00133 Rome, Italy; c National Institute for Infectious Diseases ‘L. Spallanzani’ IRCCS, Via Portuense, 292, 00149 Rome, Italy; d Freiburg Institute for Advanced Studies (FRIAS), University of Freiburg, Freiburg 79104, Germany; e The University of Manchester, Jodrell Bank Centre for Astrophysics, School of Physics and Astronomy, Manchester M13 9PL, UK; f AlgaRes, Spin off of University of Rome Tor Vergata, Via della Ricerca Scientifica 1, 00133 Rome, Italy

**Keywords:** Comparing data, Filamentous cyanobacteria, Algorithm, Deoising, Natural sample

## Abstract

The estimation and quantification of potentially toxic cyanobacteria in lakes and reservoirs are often used as a proxy of risk for water intended for human consumption and recreational activities. Here, we present data sets collected from three volcanic Italian lakes (Albano, Vico, Nemi) that present filamentous cyanobacteria strains at different environments. Presented data sets were used to estimate abundance and morphometric characteristics of potentially toxic cyanobacteria comparing manual Vs. automated estimation performed by ACQUA (“ACQUA: Automated Cyanobacterial Quantification Algorithm for toxic filamentous genera using spline curves, pattern recognition and machine learning” (Gandola et al., 2016) [1]). This strategy was used to assess the algorithm performance and to set up the denoising algorithm. Abundance and total length estimations were used for software development, to this aim we evaluated the efficiency of statistical tools and mathematical algorithms, here described. The image convolution with the Sobel filter has been chosen to denoise input images from background signals, then spline curves and least square method were used to parameterize detected filaments and to recombine crossing and interrupted sections aimed at performing precise abundances estimations and morphometric measurements.

**Specifications Table**

**Table:** 

Subject area	*Biology, Mathematics*
More specific subject area	*Image analysis*
Type of data	*Table, graph*
How data was acquired	*Microscope*
Data format	Analyzed, scatter plot, statistics
Experimental factors	Natural samples collected at −5 m depth using Niskin bottles and fixed with Lugol׳s iodine solution.
Experimental features	Samples were settled in Utermӧhl chamber and imaging was performed using Bright field microscopy.
	Abundance and total length estimation were performed with ACQUA software
Data source location	Lake Albano (41°44′50.1′′N 12°39′19.4′′E)
	Lake Nemi (41°42′27.2′′N 12°42′27.7′′E)
	Lake Vico (42°19′42.2′′N 12°10′47.6′′E)
Data accessibility	Data is within this article

**Value of the data**

• Environmental datasets and statistical analysis are shown to illustrate the development of a full-automated algorithm for filamentous cyanobacteria abundance estimation.
• Bright field images are often affected by a background color gradient and a slightly contrast, the convolution obtained by Sobel Filter represents, in just one operation, a fast computational solution for both problems.
• The parameterization of filamentous cyanobacteria through Spline curves approximated using the least square method represents a solution to recombine crossing and overlapping filaments and to perform accurate morphometric measurements and abundance evaluations.

## 1 Data

**Fig. 1:**
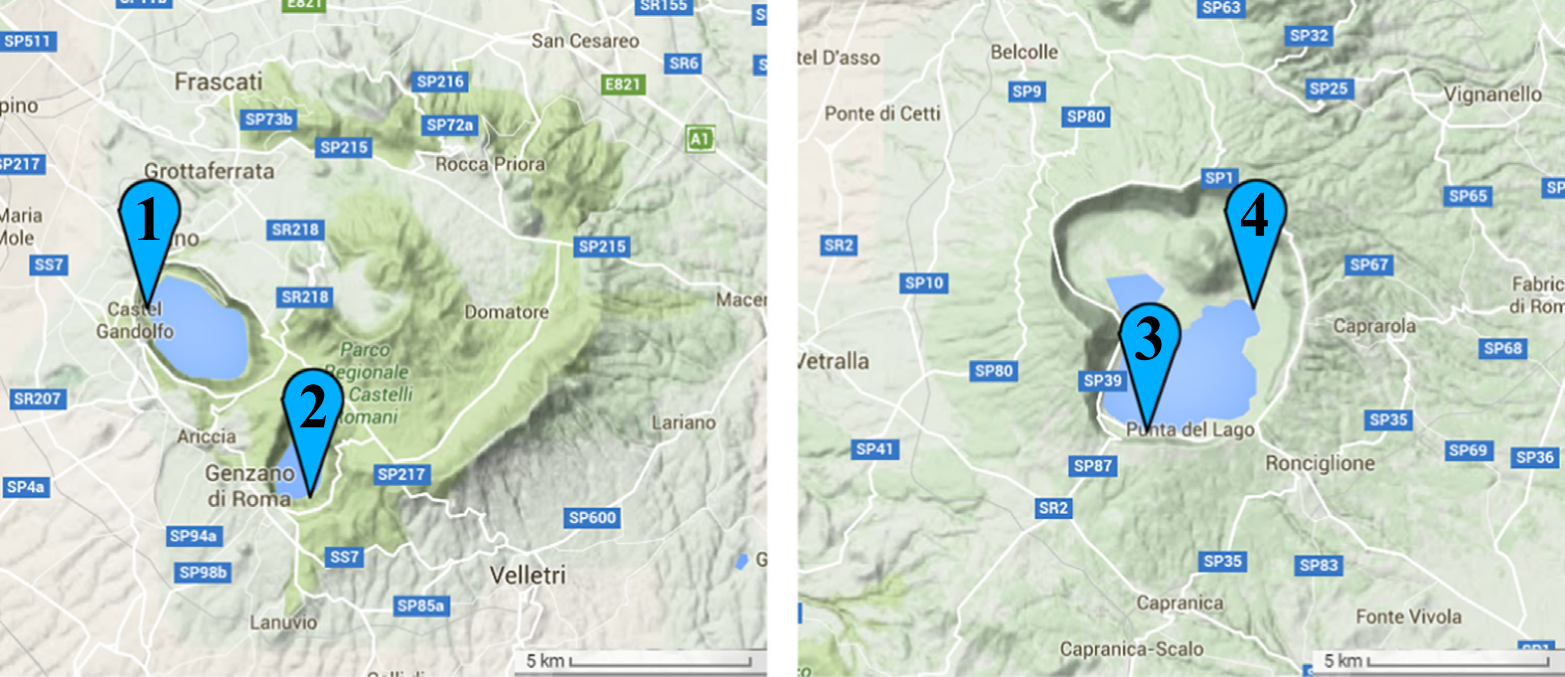
Sampling stations from 3 different Italian, Latium lakes. 1) Castel Gandolfo (Albano Lake) 2) Nemi (Nemi Lake) 3) Ronciglione (Vico Lake) 4) Caprarola (Vico Lake).

Data shown in this paper represent the evaluation datasets of ACQUA [1]. Data samples compared 4 different background condition incoming form different Italian Lakes (Fig. 1). For each water sample, 10 images were acquired and carefully manually analyzed by four independent operators. More that 500 potentially toxin cyanobacteria filaments were analyzed to compare Automatic vs Manual estimations, scatter plots and comparison tables from different data sets are shown. Mathematical methods used to perform elaboration are finally described to understand the pre-processing algorithm.

## 2 Experimental design, materials and methods

The presented experiments aim to assess the precision of ACQUA highlighting filaments of interest and evaluating abundance and total length of natural water samples. Each image was carefully recognized to obtain filament abundance, lengths, width, genus, crossing and interrupted sections and other statistical parameter. Mathematical algorithms used to this aim are also described and the implemented Matlab code is available.

(http://www.mat.uniroma2.it/~gandola/ACQU/→ACQUA_Matlab_libraries.rar).

Sobel filter convolution was used as a central operation of the pre-processing algorithm. Spline curves combined with least square method were exploited as optimal solutions to parameterize, measure and reassemble interrupted filaments.

### 2.1 Microscopy and image acquisition

Imaging was performed using a light microscope (Carl Zeiss Axiovert 100) with a 10×/0.25 objective (Achrostigmat) and a digital camera Canon 600D (18 Mpx). Image dimension was 5184×3456 pixels and resolution at 10× magnification was 0.32 μm/px. The covered area of each Field Of View (FOV) was 1.82 mm^2^.

### 2.2 Sample preparation

**Fig. 2:**
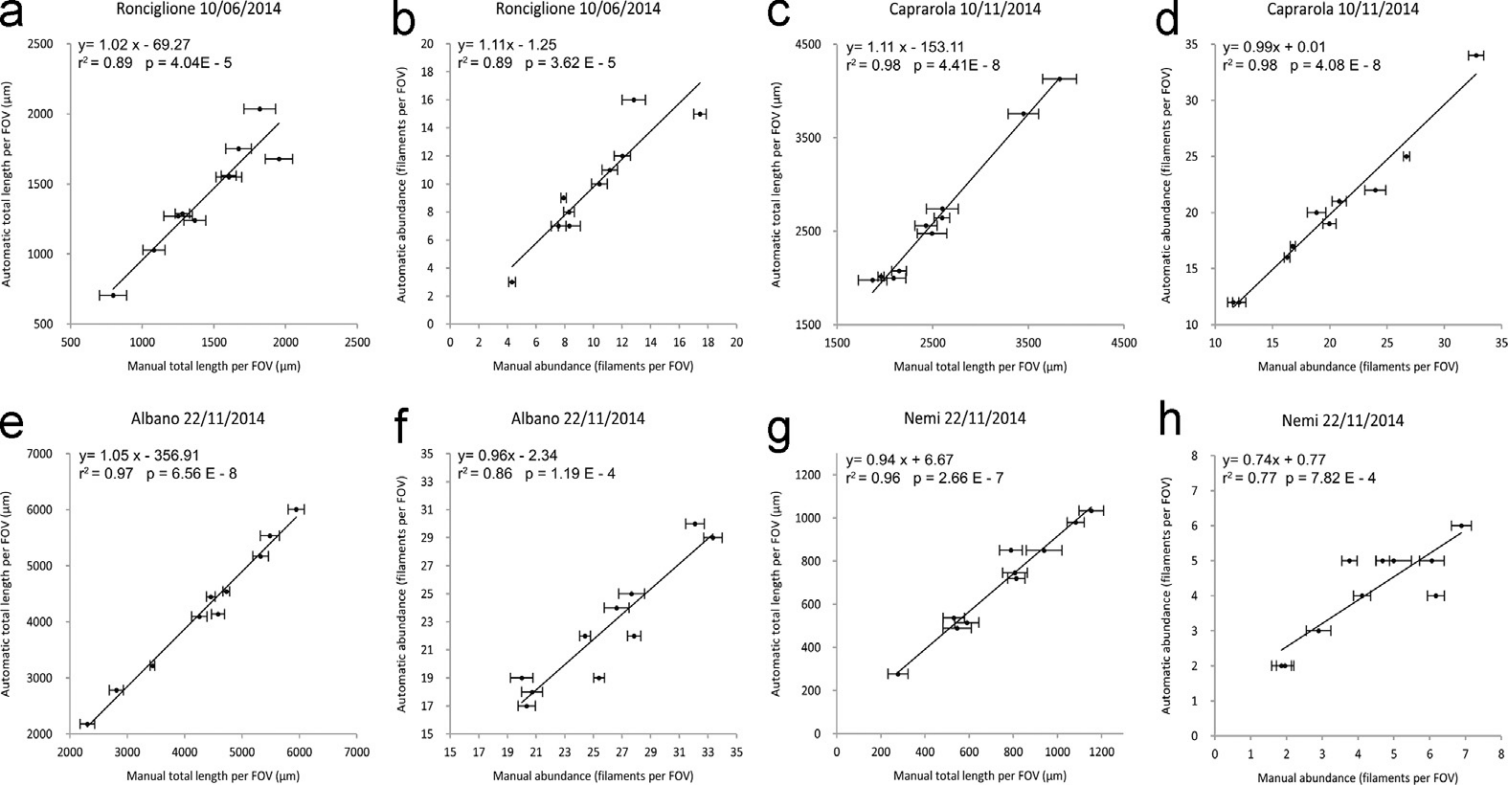
Scatter plots in Fig. 1a, c, e, g shows the comparison between automated vs. manual estimations of filament total length. Fig. 1b, d, f, h shows the correlation between automated vs*.* manual estimation of filament abundance. Regression lines are calculated through the Major Axis fitting algorithm and horizontal error bars represent the Standard deviation between manual estimation permed by four different operators.

Natural, mixed species phytoplankton samples were collected from three volcanic Italian lakes: Lake Albano, Nemi and Vico (at two stations Caprarola and Ronciglione), in June and November 2014. At each sampling station, 1 L sample was taken using a Niskin bottle, at −5 m depth and fixed with Lugol׳s iodine solution. 25 ml of subsamples were sedimented in Utermӧhl chambers (24 mm width×55 mm height) for 8 h. For each sample, 10 non-overlapping Fields Of View images were acquired. Filament abundance and total length estimation were calculated automatically by ACQUA and manually by four independent operators using the Java based software ImageJ. Image dataset is available on line at http://www.mat.uniroma2.it/~gandola/ACQUA/→Figure2DiB.rar.

### 2.3 Data set for internal validation of natural sample

Water samples were collected between June and November 2014 in four different sampling stations to collect different background noise from different lakes and seasons. Scatter plots in Fig. 2a, c, e, g show the comparison between automated and manual estimations of filament total length while graphs in Fig. 2b, d, f, h show the correlation in terms of filament abundance for each FOV. Results regarding the slope of major axis fitting lines for Caprarola, Ronciglione and Albano samples are between values of 0.96 and 1.18 that indicates a good linearity response of ACQUA estimations. In addition, *r*^2^ value is always >0.84 and p value is always <0.0001 to assess the significance of results. In the case of the Nemi sample data are biased by the presence of a large amount of background noise. To compare background noise between different samples we have calculated the percentage of interesting elements on FOVs. For example, for Caprarola abundance estimation (Fig. 2d), the average value of the total area covered by filamentous objects is 54.5% and of these 54.3% are filaments of interest (Table 1). In these conditions the correlation of the fitting line is *r*^2^=0.97 and the related slope is 0.99 (Fig. 1d). In the case of Nemi sample (Fig. 2h) only 27.4% of objects present in images were filamentous and of these just 19.1% represented elements of interest and in many cases filaments of interest were <100 µm length. Under these conditions, the *r*^2^ value is 0.72 and the slope value is 0.75. This example represents a limit case characterized by a low concentration of toxic filaments of interest and a large amount of background noise. These data confirm that the linearity of the results is confirmed in the most common conditions of natural water samples but a particular attention have to be used in presence of large amount of background noise.

Data shown in Table 1 also allow an analytical analysis of each image for any parameter measured i.e.: filament count, filament total length, number of correctly reconstructed filaments, number of total filamentous object and percentage of filamentous object area on total area covered by any elements. The 83% of broken and overlapping filaments are successfully reconstructed by ACQUA, ensuring a good estimation of both filament length estimation and abundance.

### 2.4 Convolution with Sobel Kernel

To emphasize local contrast we have chosen to perform a discrete bidimensional convolution (Formula A.1) between the input image, that is represented by the function *f* and the Sobel Kernel (Formula A.2), that is represented by the function *g*. Symbols *y* and *x* represent the height and the width of input image respectively and symbols $n_{1}$ and $n_{2}$ represent the filter indices. The Sobel Filter is composed by two different kernel (*g*_*v*_ to enlarge vertical contrast and *g*_*h*_ to magnify horizontal contrast) that requires two different passes to obtain the final recombined function *H*.

(A.1) $$h=\left(f*g\right)\left[n_{1},n_{2}\right]=\sum_{k_{1}=0}^{y}\sum_{k_{2}=0}^{x}f\left(k_{1},k_{2}\right)g\left(k_{1}-n_{1},k_{2}-n_{2}\right)$$

(A.2) $$\begin{array}{c} g_{v}=\left[\begin{array}{ccc} -1 & 0 & 1\\ -2 & 0 & 2\\ -1 & 0 & 1 \end{array}\right]\;\;\;\;\;\;\;\;\;\;\;\;\;\;\;g_{h}=\left[\begin{array}{ccc} 1 & 2 & 1\\ 0 & 0 & 0\\ -1 & -2 & -1 \end{array}\right]\;\;\;\;\;\;\;\;\;\;\;\;\;\;h_{v}=f⁎\;g_{v\;}h_{h}=\;f⁎\;g_{h}\\ H=\;\sqrt{h_{v}^{2}+{h_{h}}^{2}} \end{array}\;\;$$

**Fig. 3:**
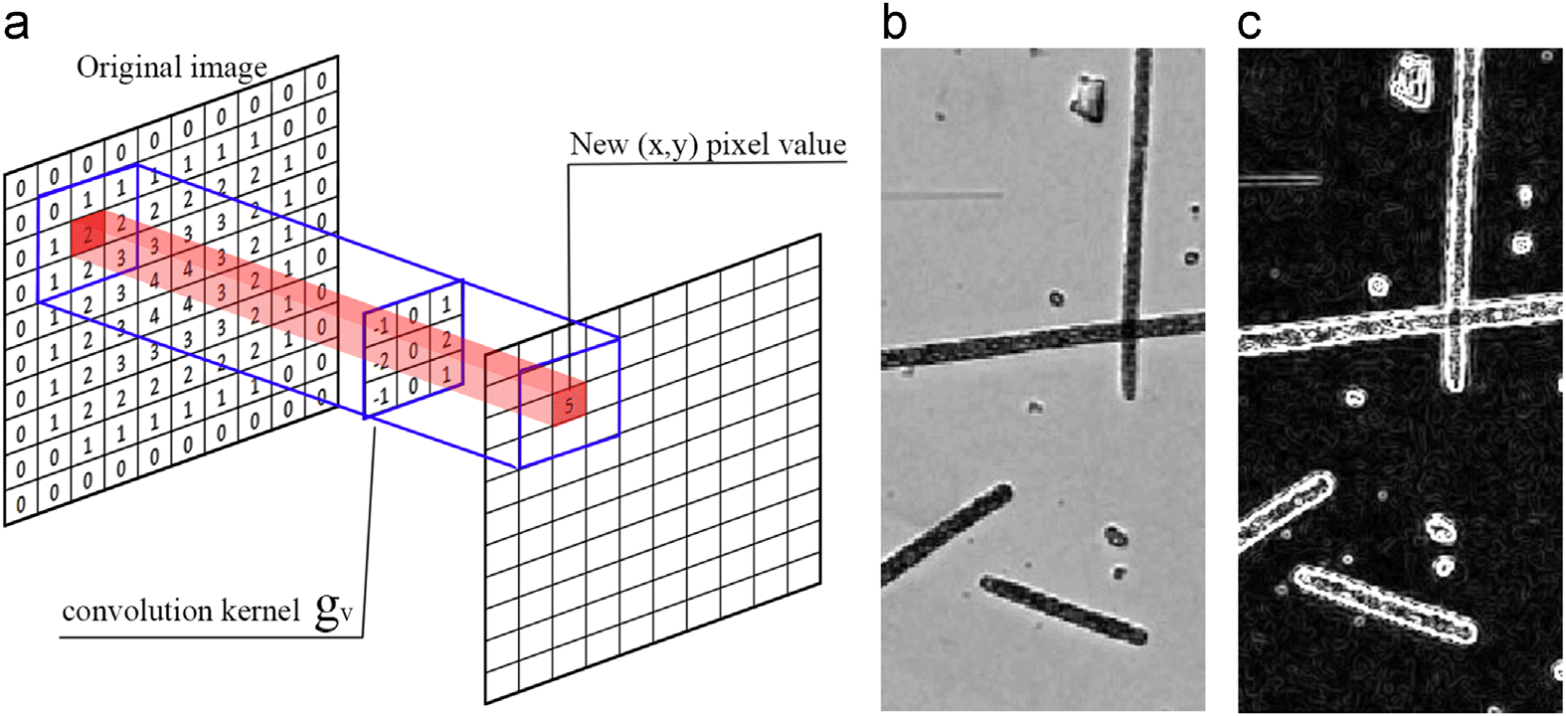
(a) Graphical illustration of a convolution between the original image and the 3×3 kernel *g*_*v.*_ (b) Original image (c) Recombined function *H*.

The result of the recombined function *H* calculated on an example image is shown in Fig. 3b and c.

## 3 Download

All developed libraries integrating the illustrated mathematical methods are available at.

http://www.mat.uniroma2.it/~gandola/ACQUA/→ACQUA_Matlab_libraries.rar.

## Figures and Tables

**Table 1:** Data for the internal validation of 40 images.

Ronciglione 10-06-2014							
Img No.	Manual Count	Manual TL (μm)	A Count	A TL (μm)	Rec fil.	Fil. obj	Afil/Aobj
1	7.6±0.5	1366±76	7	1239	2/2	39	31.4%
2	17.5±0.4	1953±96	15	1678	0/0	64	45.1%
3	8.3±0.4	1279±50	8	1288	2/2	35	31.8%
4	8.3±0.8	1249±100	7	1271	0/0	25	26.7%
5	4.3±0.2	796±95	3	705	0/0	26	23.8%
6	12.8±0.8	1671±90	16	1751	2/4	77	45.2%
7	7.9±0.2	1604±89	9	1549	0/0	43	38.9%
8	12.0±0.6	1819±110	12	2035	0/0	49	34.5%
9	10.4±0.6	1081±77	10	1029	0/0	44	33.4%
10	11.2±0.5	1603±52	11	1559	0/0	41	33.8%

Caprarola 10/11/2014							
Img No.	Manual Count	Manual TL (μm)	A Count	A TL (μm)	Rec fil.	Fil. obj	Afil/Aobj
1	11.58±0.49	1871±149	12	1976	0/0	20	63.3%
2	12.1±0.58	1958±32	12	2014	0/0	43	68.0%
3	18.85±0.81	3826±177	20	4130	5/6	29	72.7%
4	16.28±0.25	2090±128	16	1997	0/0	42	65.9%
5	16.78±0.22	2429±116	17	2560	4/4	35	69.2%
6	19.98±0.57	2148±76	19	2075	0/0	28	28.2%
7	20.83±0.62	2600±167	21	2740	0/0	42	67.0%
8	26.7±0.27	2492±155	25	2477	2/2	46	38.9%
9	32.78±0.67	3447±159	34	3756	2/2	46	41.6%
10	23.98±0.92	2596±81	22	2643	0/0	33	30.5%

Albano 22/11/2014							
Img No.	Manual Count	Manual TL (μm)	A Count	A TL (μm)	Rec fil.	Fil. obj	Afil/Aobj
1	20±0.79	4725±58	19	4543	3/4	25	79.6%
2	32.1±0.65	4582±114	30	4137	3/4	39	75.2%
3	33.35±0.65	5324±136	29	5168	2/4	36	76.5%
4	27.85±0.47	4455±76	22	4448	5/6	30	72.8%
5	20.73±0.73	2809±123	18	2783	0/0	22	57.0%
6	26.63±0.87	5944±141	24	6007	6/6	30	78.5%
7	20.35±0.6	2304±127	17	2181	2/2	29	64.6%
8	25.4±0.37	3438±41	19	3218	0/0	27	69.0%
9	27.68±0.9	5485±165	25	5535	4/5	27	78.9%
10	24.43±0.38	4256±134	22	4093	0/0	25	80.0%

Nemi 22/11/2014							
Img No.	Manual Count	Manual TL (μm)	A Count	A TL (μm)	Rec fil.	Fil. obj	Afil/Aobj
1	6.888±0.28	1083±39	6	980	0/0	17	27.2%
2	5±0.49	546±64	5	489	0/0	24	26.2%
3	4.113±0.24	589±55	4	515	0/0	10	18.4%
4	4.688±0.19	808±56	5	746	0/0	20	26.5%
5	1.963±0.24	531±49	2	537	0/0	10	23.3%
6	6.063±0.34	940±81	5	850	0/0	23	29.5%
7	6.175±0.24	814±39	4	720	0/0	26	30.2%
8	1.863±0.28	278±45	2	277	0/0	23	27.2%
9	2.9±0.34	789±51	3	850	0/0	44	37.7%
10	3.763±0.21	1154±56	5	1034	0/0	18	28.2%

Manual and automated count and filament length are given, and typical issues such as reconstructed filaments, total filamentous objects in FOV and percentage of filament elements to noise are quantified.
